# Awaited paradigm shift in marine N_2_ fixing ecology

**DOI:** 10.1093/nsr/nwaf415

**Published:** 2025-09-25

**Authors:** Keisuke Inomura

**Affiliations:** Graduate School of Oceanography, University of Rhode Island, USA

*Crocosphaera watsonii* (hereafter, *Crocosphaera*—note that, nowadays, this term technically includes broader taxa [1], but here it is used for UCYN-B and *Crocosphaera watsonii*, Fig. 1) has been thought of as one of the key nitrogen fixers in the surface ocean. However, its global impact has been overshadowed by other N_2_ fixers, including *Trichodesmium, Richelia* and UCYN-A. For example, a recent review shows a map with limited *Crocosphaera* habitats [2], following earlier data compilation [3]. A machine-learning approach shows relatively small areas of *Crocosphaera* domination [4] and a recent metagenomic analysis shows that *Crocosphaera* did not dominate in any size classes [5].

**Figure 1.**
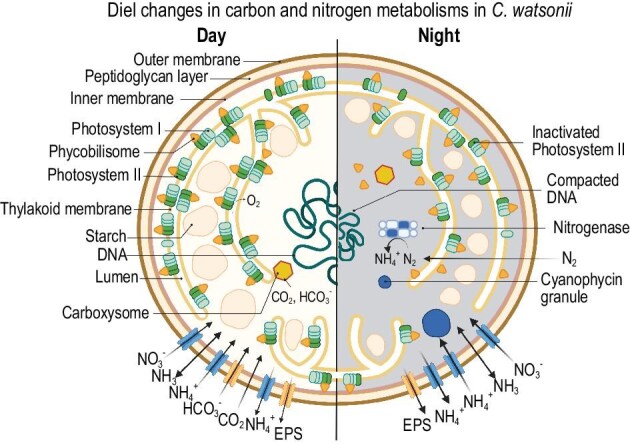
Schematics of UCYN-B or *Crocosphaera watsonii* cell and its day–night metabolic shift. Figure is adapted from Masuda *et al.* (2022) [12] under license number CC BY 4.0.

At the same time, predictions and evidence for the high abundance of *Crocosphaera* did exist. An ecological simulation showed *Crocosphaera* analogue dominating in the North Pacific Ocean with effective iron utilization [6]. Although not genomically confirmed as *Crocosphaera*, a high abundance of nanoplanktonic cyanobacteria and their close correlation with the rate of N_2_ fixation were observed in the North Pacific gyre [7] with a subsequent study having shown an even bigger niche of nanoplanktonic cyanobacteria, likely *Crocosphaera* [8]. The biomass of *Crocosphaera* was estimated to be substantially larger than those of even major non-N_2_-fixing cyanobacteria, such as *Prochlorococcus* and *Synechococcus*, at a sampling point in the North Western Pacific [9]. However, deterministic evidence that demonstrated a wide area of *Crocosphaera* dominance was still missing.

Jiang *et al*. [10], together with a previous work from a similar region [11], convincingly shows that *Crocosphaera* dominates in an extended region in the Subtropical North Pacific, with measurements of the *nifH* gene abundance of major cyanobacterial diazotrophs. The results show that *Crocosphaera* dominates across a range of 10 degrees in latitude and 30 degrees in longitude—arguably the widest region of *Crocosphaera* domination observed to date in genomics studies. Jiang *et al*. [10] added these new data to the global dataset of N_2_ fixers and ran a statistical model that showed a *Crocosphaera* niche beyond the observed area in the present study. These areas include the Southern Hemispheric Indian Ocean and the Central South Pacific Subtropical gyre. These predictions guarantee the importance of observations in these regions to test the dominance of *Crocosphaera* and the rate of N_2_ fixation.

However, the mechanics of *Crocosphaera* domination still remain elusive. Useful information has been provided by the present study [10], in which the abundance of different N_2_ fixers is related to three key factors: temperature, Fe and phosphate. Yet, challenges still remain because (i) there are overlapping relationships across taxa and (ii) these relationships provide limited ecophysiological interpretation. For example, regarding (i), there is an overlapping abundance–Fe relationship between *Crocosphaera* and *Trichodesmium*. Regarding (ii), an increasing abundance of *Crocosphaera* is associated with decreasing Fe above a certain threshold, but why is that the case? How does the unique diurnal metabolic cycle (Fig. 1 and [6,12]) support their niche? How does the heterogeneous metabolic population (i.e. mixture of N_2_-fixing and non-N_2_-fixing cells [13]) contribute to their regional dominance? A multi-methodological approach [14], synthesizing modeling and experiments, is essential in addressing such mechanistic uncertainties.

***Conflict of interest statement.*** None declared.

## References

[1] Masuda T, Mareš J, Shiozaki T et al. J Phycol 2024; 60: 604–20 10.1111/jpy.13450 38551849

[2] Zehr JP, Capone DG. Science 2020; 368: eaay9514. 10.1126/science.aay9514 32409447

[3] Luo YW, Doney SC, Anderson LA et al. Earth Syst Sci Data 2012; 4: 47–73. 10.5194/essd-4-47-2012

[4] Tang W, Cassar N. Geophys Res Lett 2019; 46: 12258–69. 10.1029/2019GL084376

[5] Pierella Karlusich JJ, Pelletier E, Lombard F et al. Nat Commun 2021; 12: 4160. 10.1038/s41467-021-24299-y 34230473 PMC8260585

[6] Saito MA, Bertrand EM, Dutkiewicz S et al. Proc Natl Acad Sci USA 2011; 108: 2184–9. 10.1073/pnas.1006943108 21248230 PMC3038740

[7] Kitajima S, Furuya K, Hashihama F et al. Limnol Oceanogr 2009; 54: 537–47. 10.4319/lo.2009.54.2.0537

[8] Sato M, Hashihama F, Kitajima S et al. Aquat Microb Ecol 2010; 59: 273–82. 10.3354/ame01397

[9] Masuda T, Inomura K, Kodama T et al. Microbiol Spectr 2022; 10: e02177–21. 10.1128/spectrum.02177-21 35770981 PMC9431459

[10] Jiang R, Hong H, Wen Z et al. Natl Sci Rev 2025; 12: nwaf337. 10.1093/nsr/nwaf337 41040495 PMC12485613

[11] Wen Z, Browning TJ, Cai Y et al. Sci Adv 2022; 8: eabl7564. 10.1126/sciadv.abl7564 35119922 PMC8816331

[12] Masuda T, Inomura K, Mareš J et al. Trends Microbiol 2022; 30: 805–6. 10.1016/j.tim.2022.02.006 35331632

[13] Masuda T, Inomura K, Takahata N et al. Commun Biol 2020; 3: 172. 10.1038/s42003-020-0894-4 32286494 PMC7156374

[14] Inomura K, Deutsch C, Masuda T et al. Comput Struct Biotechnol J 2020; 18: 3905–24. 10.1016/j.csbj.2020.11.022 33335688 PMC7733014

